# Habenula activation patterns in a preclinical model of neuropathic pain accompanied by depressive-like behaviour

**DOI:** 10.1371/journal.pone.0271295

**Published:** 2022-07-12

**Authors:** Geiza Fernanda Antunes, Ana Carolina Pinheiro Campos, Danielle Varin de Assis, Flavia Venetucci Gouveia, Midiã Dias de Jesus Seno, Rosana Lima Pagano, Raquel Chacon Ruiz Martinez

**Affiliations:** 1 Division of Neuroscience, Hospital Sírio-Libanês, São Paulo, Brazil; 2 Neuroscience and Mental Health, Hospital for Sick Children Research Institute, Toronto, Canada; 3 LIM/23, Institute of Psychiatry, University of Sao Paulo School of Medicine, São Paulo, Brazil; University of Minho, PORTUGAL

## Abstract

Pain and depression are complex disorders that frequently co-occur, resulting in diminished quality of life. The habenula is an epithalamic structure considered to play a pivotal role in the neurocircuitry of both pain and depression. The habenula can be divided into two major areas, the lateral and medial habenula, that can be further subdivided, resulting in 6 main subregions. Here, we investigated habenula activation patterns in a rat model of neuropathic pain with accompanying depressive-like behaviour. Wistar rats received active surgery for the development of neuropathic pain (chronic constriction injury of the sciatic nerve; CCI), sham surgery (surgical control), or no surgery (behavioural control). All animals were evaluated for mechanical nociceptive threshold using the paw pressure test and depressive-like behaviour using the forced swimming test, followed by evaluation of the immunoreactivity to cFos—a marker of neuronal activity—in the habenula and subregions. The Open Field Test was used to evaluate locomotor activity. Animals with peripheral neuropathy (CCI) showed decreased mechanical nociceptive threshold and increased depressive-like behaviour compared to control groups. The CCI group presented decreased cFos immunoreactivity in the total habenula, total lateral habenula and lateral habenula subregions, compared to controls. No difference was found in cFos immunoreactivity in the total medial habenula, however when evaluating the subregions of the medial habenula, we observed distinct activation patterns, with increase cFos immunoreactivity in the superior subregion and decrease in the central subregion. Taken together, our data suggest an involvement of the habenula in neuropathic pain and accompanying depressive-like behaviour.

## Introduction

Neuropathic pain is a disorder with a prevalence of 7–10% [1] that results in great suffering in patients and a significant burden to the healthcare system [2]. Similarly, depression affects approximately 6% of adults worldwide [3]. Interestingly, patients with debilitating pain often present with depressive symptoms [4–6], while individuals who are depressed also demonstrate an exacerbated pain perception [7, 8]. Hence, pain-associated depression refers to a complex disorder in which persistent pain and major depressive disorder co-occur, displaying synergic symptoms [9, 10]. Because of this synergism, pain and depression treatments rely on similar mechanisms [11].

It has been proposed that the neuroanatomical bases of pain and depression involve similar brain areas, such as the thalamus, amygdala and habenula [12–16]. The thalamus is a key area in the ascending pain pathway (i.e. spinothalamic tract) and also thought to be critically involved in depressive symptoms [14, 17]. Distinct amygdala subregions have been shown to be involved in the expression of depressive-like behaviours in rodent models of neuropathic pain [15]. Specifically, the anterior and posterior portions of the basolateral nucleus of the amygdala (BLA) and the central portion of the central nucleus of the amygdala (CeA) are involved in the neurocircuitry underlying neuropathic pain and the pharmacological inactivation of these areas reverses hyperalgesia, allodynia and depressive-like behavior in animals with peripheral neuropathy [15].

The habenula (Hb), an epithalamic structure of the limbic system, plays a key role in the endocrine system, reward, addiction, pain, and depressive behaviours [12, 13, 18]. The Hb can be divided into lateral (LHb) and medial (MHb) parts, based on cell type and connectivity pattern [19, 20]. The LHb can be further subdivided into lateral (LHbL) and medial (LHbM) regions, while the MHb can be parcellated into superior (MHbS), inferior (MHbI), central (MHbC), and lateral (MHbL) regions [20–23]. The LHb sends several outputs to the raphe nuclei and ventral tegmental area [24], which are strongly related to analgesia [25] and have been proposed to play a prominent role in pain processing [26, 27], and in depressive-like behaviours in models of neuropathic pain [15, 28, 29]. Increased LHb activity is associated with depressive behaviours [30, 31] via increased GABAergic neurotransmission, resulting in inhibition of the dopaminergic and serotonergic systems [32, 33], which are involved in mechanisms of pain and depressive symptoms [28]. Furthermore, pharmacological inhibition of the lateral habenula improves depressive-like behaviour in a rat model of depression [16].

The MHb has been implicated in stress, depression, memory processing, and nicotine withdrawal syndrome [30, 34–36] and has a potential role in pain control [37]. Kim and Chang (2005) suggested that MHb may mediate LHb activity by “*boutons en passant”* synapses from the MHb to the LHb [38]. The MHb is believed to receive inputs from different areas within the limbic system and projects to the interpeduncular nucleus (IPN), which in turn projects to specific areas of the limbic system, thought to be involved in both pain and depressive behaviours [19, 39], including the serotonergic raphe nuclei [40, 41]. The IPN receives input from the MHbS via substance P and from the MHbI via acetylcholine [42–45]. High levels of mu opioid receptor (MOR) can be found in cholinergic neurons in the MHb [46, 47] and are also distributed along the MHb-IPN pathway, co-localizing with substance P [48]. Interestingly, elevated expression of substance P was observed in the MHb-IPN connection in animals presenting depressive-like behaviours, and MHb lesions were sufficient to suppress these behaviours [49].

In this study, we aimed to investigate the activation pattern of the LHb and MHb and its subareas (LHbL, LHbM, MHbS, MHbI, MHbC, and MHbL) in a preclinical rat model of neuropathic pain accompanied by depressive-like behaviour.

## Materials and methods

### Animals

Seventeen male Wistar rats (200–250 g) were used in this study. All animals were maintained in regular rat cages (2–3 rats/box) with wood shavings and free access to water and rat chow pellets under a 12h light/dark cycle and controlled temperature (22±2°C). Before the experimental procedures, animals were allowed to habituate to the animal facility for one week. The protocols used in this project were approved by the Ethics Committee on the Use of Animals for Research of the Hospital Sírio-Libanês (Brazil, CEUA# 2014/07) and were conducted and reported in accordance with the ARRIVE guidelines (http://www.nc3rs.org.uk/arrive-guidelines).

### Experimental design

**Fig 1:**
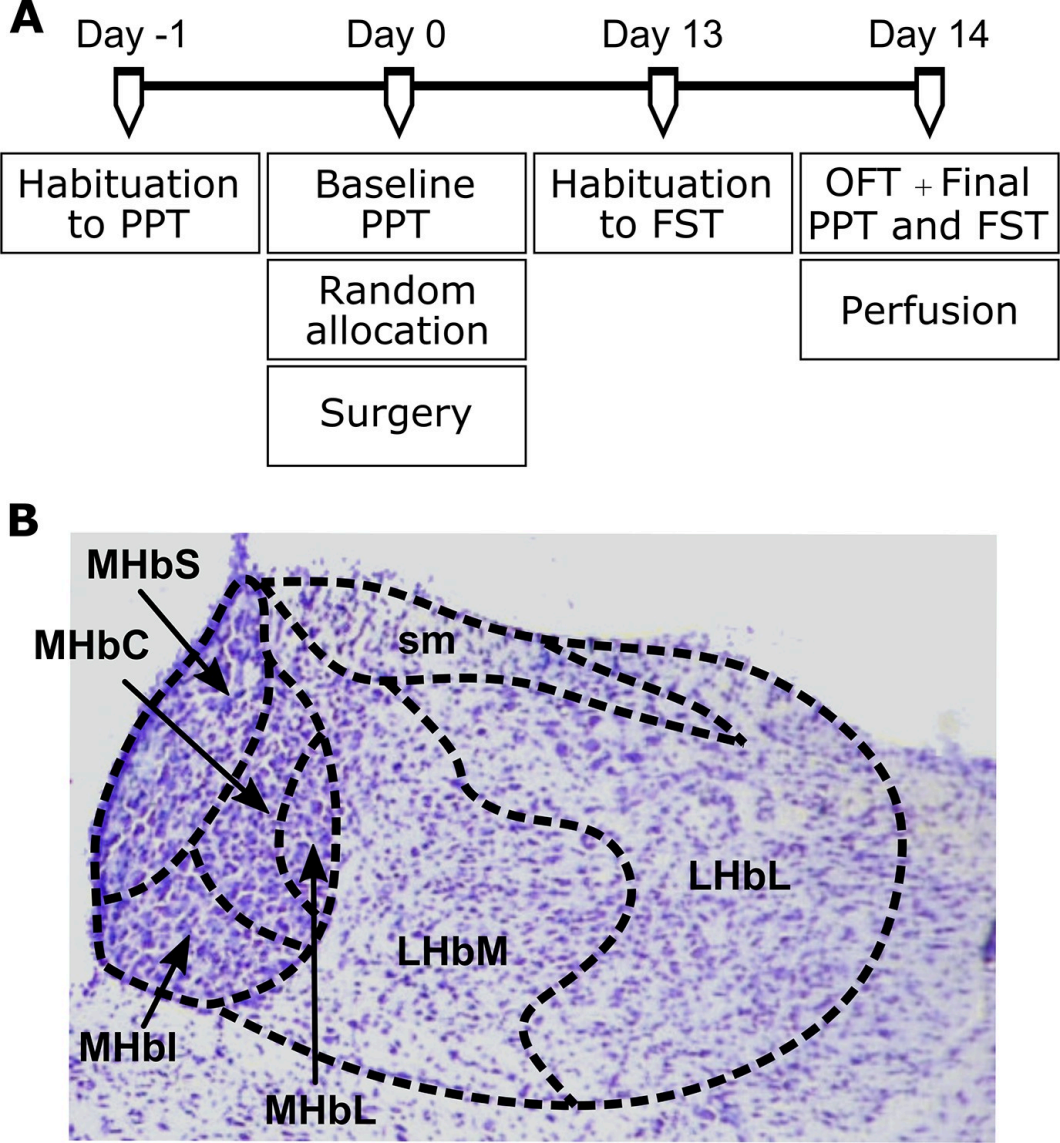
Methods of study. A. Experimental design. After habituation to the animal facility, Wistar rats habituated to the paw pressure test (PPT) and, on the following day, were evaluated for baseline measures of mechanical nociceptive threshold. Animals were then randomly allocated into three groups (i.e. naive [no surgery], false-operated, and chronic constriction injury [active surgery]), followed by the assigned surgery. Thirteen days after the baseline measure, animals were habituated to the Forced Swimming Test (FST). On the following day, animals were tested in the Open Field Test (OFT) and final measures of the PPT and FST were taken. B. Photomicrography of a Nissl-stained coronal slice, showing the lateral and medial habenula and its subdivisions. Abbreviations: LHbL: Lateral subdivision of the lateral habenula. LHbM: Medial subdivision of the lateral habenula. MHbS: Superior subdivision of the medial habenula. MHbI: Inferior subdivision of the medial habenula. MHbL: Lateral subdivision of the medial habenula. MHbC: Central subdivision of the medial habenula. sm: stria medullaris.

After habituation to the animal facility, animals were habituated to the paw pressure test (PPT) apparatus (10 minutes). On the next day, baseline measures of mechanical nociceptive thresholds were obtained for all animals, followed by random allocation into three groups: I. naive (n = 6, no surgery); II. false-operated (FOP, n = 6, sham surgery); and III. chronic constriction injury (CCI, n = 5; active surgery) and surgery. After 13 days, animals were habituated to the forced swimming test (FST). On the last day (day 14 after surgery) all animals were evaluated in the Open Field Test (OFT), PPT and FST. Ninety minutes after behavioural tests, the animals were transcardially perfused, and brains were recovered for histological analysis. Fig 1A illustrates the study timeline.

### Peripheral neuropathy surgery

The CCI model was established as previously described [15, 50, 51]. Briefly, rats were anaesthetised with Isoflurane (4–5% induction, 2–3% maintenance), the right sciatic nerve was exposed, and four ligatures (1–1.5 mm apart) were loosely tied around the nerve using 4.0 Catgut chromic sutures. FOP rats were anaesthetised, and the right sciatic nerve was exposed, but there was no constriction of the nerve. Naive rats received no surgery.

### Evaluation of nociceptive threshold–Paw pressure test

The mechanical nociceptive threshold was determined using a PPT apparatus (EEF-440, Insight, SP, Brazil), as previously described [52]. Briefly, the hind paw of the animal was placed into the apparatus, and the force (in grams) required to induce a paw withdrawal response represented the mechanical nociceptive threshold. All animals were habituated to the apparatus before testing, by handling the animals and simulating the test without applying paw pressure. The PPT was conducted on all animals at baseline and last time point. A significant reduction in mechanical nociceptive thresholds represented neuropathic pain.

### Evaluation of depressive-like behaviour–Forced swimming test

Depressive-like behaviour was determined using the FST, as previously described [53]. A cylindrical tank (30 cm diameter × 60 cm height) was filled with 30 cm high lukewarm water (24±1°C), and animals were gently placed on the water. All animals were habituated to the FST for 15 minutes one day before testing. On the last day, all animals were tested in the FST for 5 minutes. Immobility time (in seconds) was determined by measuring the time during which no additional activity was observed other than the movements necessary to keep the head above the water surface. Increased time spent immobile characterized depressive-like behaviour.

### Evaluation of locomotor activity–Open Field Test

The OFT was used to evaluate locomotor behaviour as a control for possible locomotor impairment that could confound the results of the remaining behavioural tests. The PFT apparatus consists of a 60x60x50 dark grey Formica box. No habituation to the test is required. During the test, each animal was placed in the centre of the apparatus and allowed to freely explore for 5 min. The behaviour was video-recorded and the total distance travelled during the test was evaluated by a blind observer. After the end of the test, the open field was cleaned with 5% ethanol and subsequently dried with a cloth

### Histological analysis–Immunohistochemistry for cFos and Nissl-staining

The immunohistochemistry (IHC) protocol was performed as previously described [15, 51]. Briefly, brains were frozen cut in sequential 30μm-thick slices. Brain slices were then washed in buffer and incubated overnight at 4°C with rabbit anti- cFos primary antibody (1:20000; Ab-5, Calbiochem, CA, USA) followed by incubation with biotinylated secondary antibody (1:200; donkey anti-rabbit IgG, Jackson ImmunoResearch, PA, USA) and avidin-biotin complex (1:100; ABC Elite kit, Vector Laboratories, CA, USA). The antibody complex was visualized by exposure to a chromogen solution containing 0.05% diaminobenzidine tetrahydrochloride (DAB, Sigma-Aldrich, MO, USA) and 0.01% hydrogen peroxide in the buffer. Images were captured using a light microscope (E1000, Nikon, NY, USA), and cFos immunoreactivity (cFos-IR) of the Hb was evaluated by a blinded observer at 10x magnification. Adjoining Nissl-stained sections provided the histological landmarks for the accurate identification and delineation of the LHb (LHbL and LHbM) and of the MHb (MHbS, MHbI, MHbC, MHbL) regions (Bregmas: -3.00 mm to -4.36 mm of the Paxinos and Watson Atlas [54]; Fig 1B).

### Statistical analyses

Data are presented as the mean ± standard error of the mean (SEM). Statistical analyses were conducted using GraphPad Prism software (version 5.0; GraphPad Software Inc., CA, USA). Normal distribution was confirmed for all variables using the Kolmogorov-Smirnov test. Mechanical nociceptive thresholds were analyzed with two-way repeated measures analysis of variance (ANOVA), followed by Tukey’s post-hoc test. Immobility time in the FST and total distance travelled in the OFT were evaluated with one-way ANOVA, followed by Tukey’s post-hoc test, where applicable. cFos-IR was normalized by defining the naive group as 100%, and analyzed using one-way ANOVA, followed by Tukey’s post-hoc test. For all tests, statistical significance was set at p<0.05. Power analysis was performed to assess the power of this study, as previously described [55]. Considering the mechanical nociceptive threshold in the PPT the primary outcome measure, and power (1-β) set at 0.80 (i.e. 80% power) and α = 0.05 (i.e. significance level of p<0.05), the analysis resulted in a minimum sample size of 4.2 animals per/group.

## Results

**Fig 2:**
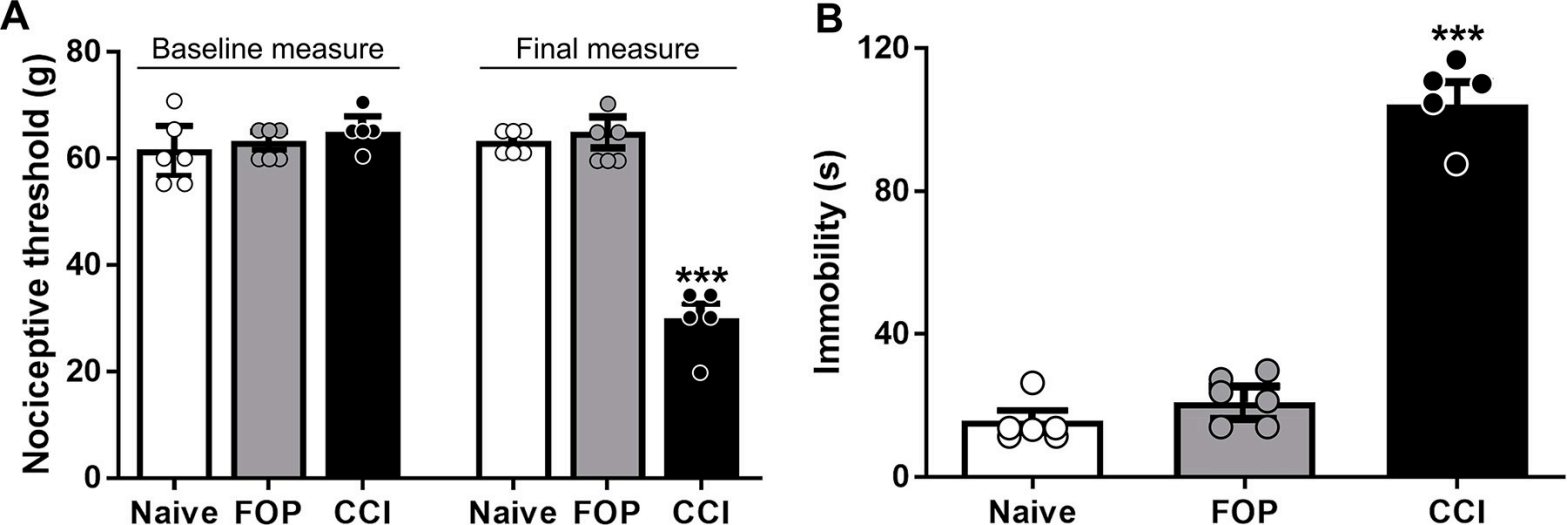
Behavioural results. A. Mechanical Nociceptive Threshold (g) in the paw pressure test before randomization (baseline measure) and after 14 days (final measure). A significant reduction in mechanical nociceptive thresholds represents neuropathic pain. B. Immobility time (s) in the Forced Swimming Test, evaluated 14 days after group allocation. Increased time spent immobile characterized depressive-like behaviour. Values are presented as mean ± SEM. ***p < 0.001. Abbreviations: FOP: false-operated, CCI: chronic constriction injury.

The CCI group showed a significant decrease in the mechanical nociceptive threshold at the final measurement (F_(2,14)_ = 29.92, p<0.001, Fig 2A, Table 1) and a significant increase in immobility time in the FST (F_(2,12)_ = 114.8, p<0.001, Fig 2B, Table 1), compared to control groups. No differences between groups was observed in the total distance travelled in the OFT (F_(2,14)_ = 0.998, p>0.05, Table 1).

**Table 1:** Mean and standard deviation—behaviour and habenula c-fos immunoreactivity.

Variable	Naive	FOP	CCI
**Paw Pressure Test**	60.83±5.85	63.33±4.08	30.00±6.12***
**Forced Swimming Test**	15.50±5.68	19.00±7.75	92.80±27.96***
**Open Field Test**	731.67±82.80	841.67±145.66	758.00±183.36
**Total Habenula**	100±12.5	64.5±30.4	55±12*
**Total Lateral Habenula**	100±8.4	45.7±13.9	33.2±13.8**
**Lateral Habenula–Lateral**	100±13.2	43.6±16.1	33.4±15.3*
**Lateral Habenula—Medial**	100±7.4	49.9±12.8	29.4±14.5*
**Total Medial Habenula**	100±24.8	84.5±40.1	79.9±15.6
**Medial Habenula—Superior**	100±26.5	222±60.4	454.2±65*
**Medial Habenula—Central**	100±12.3	82.7±22.8	53.4±5.4***
**Medial Habenula—Lateral**	100±15.7	76.5±20.7	93.8±8.7
**Medial Habenula—Inferior**	100±35.9	81.6±8.82	44.0±7.34

Abbreviations: FOP: false-operated, CCI: chronic constriction injury

*p<0.05

**p<0.01

***p<0.001

**Fig 3:**
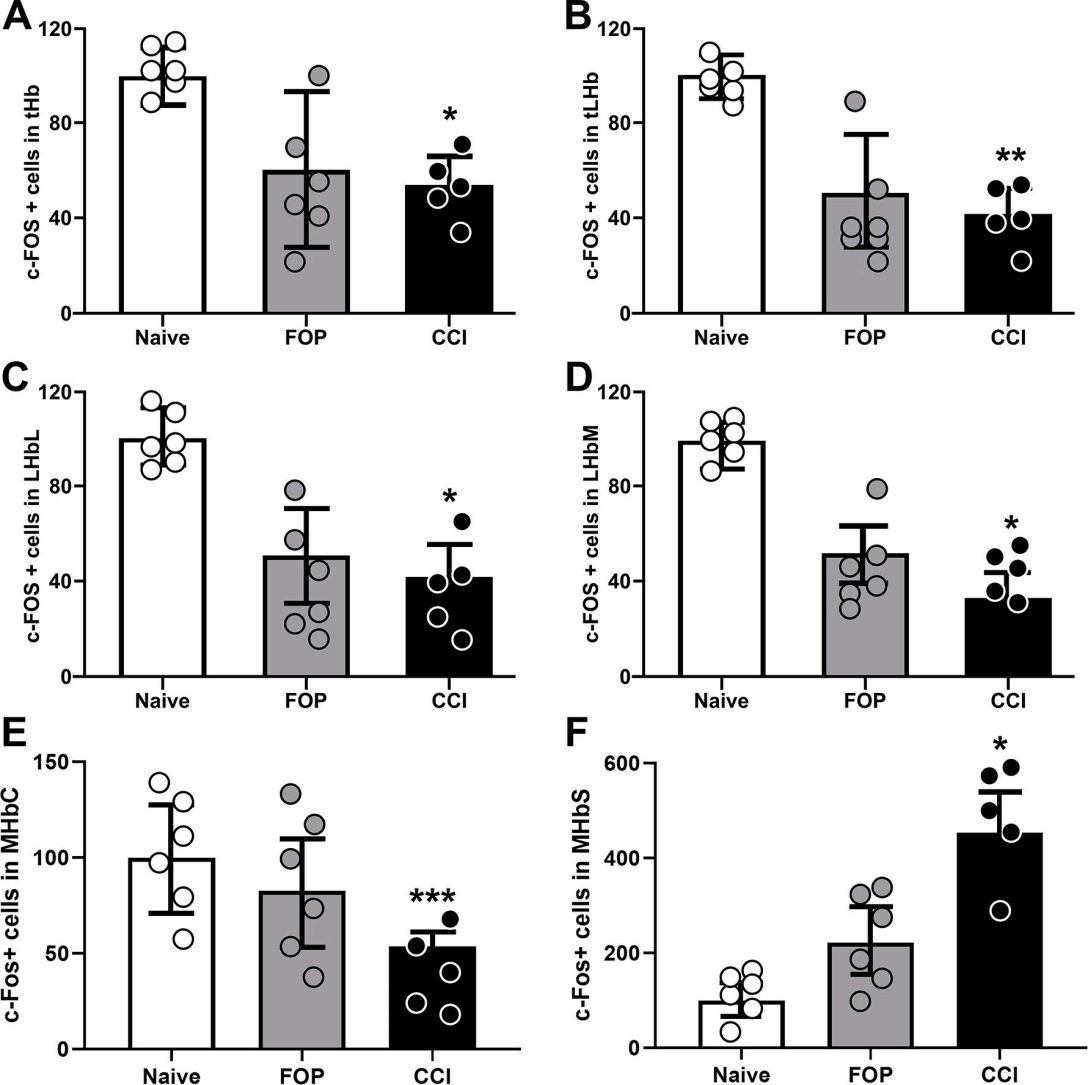
cFos immunoreactivity (cFos-IR) pattern in the habenula. A. Total Habenula (tHb) cFos-IR. B. Total Lateral Habenula (tLHb) cFos-IR. C. cFos-IR in the lateral subregion of the LHb (LHbL). D. cFos-IR in the medial subregion of the LHb (LHbM). E. cFos-IR in the central subregion of the medial habenula (MHbC). F. cFos-IR in the superior subregion of the medial habenula (MHbS). Abbreviations: FOP: false-operated group, CCI: chronic constriction injury group. Values are presented as normalized mean±SEM. *p<0.05, **p<0.01, ***p<0.001.

A significant reduction in cFos-IR were observed in the CCI group in the total Hb (F_(2,14)_ = 4.696, p<0.05, Fig 3A, Table 1) when compared to naive controls. There was also a significant reduction in cFos-IR in the total LHb (F_(2,14)_ = 7.032, p<0.01, Fig 3B, Table 1), subregions LHbL (F_(2,14)_ = 4.512, p<0.05, Fig 3C, Table 1), LHbM (F_(2,14)_ = 4.248, p<0.05, Fig 3D, Table 1), in the CCI group compared to FOP and naive groups. When evaluating the sub-regions of the MHb, we observed a significant reduction in cFos-IR in the MHbC (F_(2,12)_ = 17.84, p = 0.0011, Fig 3E, Table 1) and significant increase in cFos-IR in the subregion MHbS (F_(2,13)_ = 6.574, p = 0.013, Fig 3F, Table 1), in the CCI group compared to FOP and naive groups. No differences in cFos-IR were observed between groups when evaluating total MHb (F_(2,11)_ = 0.326, p = 0.72, Table 1), subregion MHbL (F_(2,14)_ = 0.611, p = 0.55, Table 1) and subregion MHbI (F_(2,13)_ = 0.087, p = 0.91; Table 1).

## Discussion

In this study we described the activation pattern of the Hb, and its subregions, in a preclinical neuropathic pain model accompanied by depressive-like behaviour. Clinical and preclinical studies have provided evidence of the involvement of the Hb in pain and depressive behaviours. Using functional magnetic resonance imaging, (fMRI), Shelton and colleagues (2012) showed bilateral Hb activation during noxious stimulation, suggesting the Hb to be involved in the pain processing network [27]. In an examination of transient effects of deep brain stimulation in the Hb, Zhang and colleagues showed that one of the most common transient effects associated with increased voltage was pain [56]. Preclinical studies have demonstrated that the electrical stimulation of the Hb, or intra-nuclear morphine injections have been shown to induce analgesia [57, 58], while lesions restricted to the MHb, to the IPN, or to the fibre bundle connecting these structures, increase pain sensitivity [59]. Furthermore, significant reductions in LHb activation patterns were also observed in animal models of diabetes-induced neuropathic pain [60] and tail pinch intermittent stressor [61]. In line with these findings, in this study we observed a significant reduction in cFos-IR in the LHb and subdivisions in animals with neuropathic pain accompanied by depressive-like behaviours. It is important to highlight that with our methodology, we are able to determine the stimulus-induced nociception at day 14 post surgery, but not to perform an ongoing evaluation of pain throughout the study [62, 63]. Also, the increased time in immobility observed in the CCI group is not a result of impaired locomotor activity, as no differences were observed between groups in the OFT. As both pain and depression are observed simultaneously, it is not possible to depict which component is more relevant for the cFos expression pattern observed in the Hb. A study focusing on pharmacological manipulations (e.g. use of antidepressants) could provide some insight into this aspect.

It has been shown that patients diagnosed with major depressive disorder [64] and in psychiatric disorders that present depressive components [65, 66] present with altered habenula volume. Furthermore, deep brain stimulation of the habenula results in symptom alleviation in depressive patients [67, 68]. It has been shown that the LHb is involved in effort-based decision-making, a key contributor to willingness to exert physical effort in psychiatric conditions [69]. Han and colleagues (2017) showed a down-regulation of cholinergic genes in the Hb of animals exposed to the chronic restraint stress model of depression [70]. Previous studies have also shown increased metabolic activity in the MHb of a genetic rat model of helpless behaviour [30] and rats exposed to the chronic unpredictable mild stress paradigm [49].

Although we did not observe a significant difference between groups in cFos-IR of the total MHb, when analyzing its subregions, we noted a distinct activation pattern, with the CCI group presenting increase cFos-IR in the MHbS and reduced cFos-IR in the MHbC. These results suggest heterogeneity in the MHb subregions and highlight the importance of further investigating the role of MHb subareas in depressive behaviours. While the MHbS consists exclusively of densely packed glutamatergic neurons that strongly express interleukin-18, MHbC is composed of neurons that either co-express substance-P and glutamate, or acetylcholine and glutamate [21, 71]. Efferents from the MHb forms the core aspect of the fasciculus retroflexus, with dorsal projections reaching lateral aspects of the IPN, medial projections reaching the ventral aspect of IPN and lateral projections ending on the dorsal aspect of IPN [19]. It is believed that glutamatergic projections from the MHb terminate in the IPN, cholinergic and substance P-ergic projections through the IPN and indirect connections terminate in the VTA, and additional projections from the MHb reaches the raphe nuclei and LHb [38, 72]. These connections suggest a possible modulatory role of both the MHbS and MHbC on serotonin, IPN and LHb function, and of the MHbC on dopamine [72]. This work sheds light on the involvement of the Hb in the neural-network of neuropathic pain and accompanying depressive-like behaviour. Further studies are necessary to better understand the neurobiological mechanisms underlying neuropathic pain and depression.
